# Immunomodulatory role of EV-derived non-coding RNA in lung cancer

**DOI:** 10.20517/evcna.2022.42

**Published:** 2023-03-24

**Authors:** Patrizia Ghidotti, Ilaria Petraroia, Orazio Fortunato, Francesca Pontis

**Affiliations:** Tumor Genomics Unit, Fondazione IRCCS Istituto Nazionale dei Tumori, Milan 20133, Italy.

**Keywords:** Extracellular vesicles, microRNA, lung cancer, immune cells

## Abstract

Lung cancer is the deadliest cancer worldwide, primarily because of its metastatic spread. Extracellular vesicles (EVs) are small lipid-bilayer particles released by almost all types of cells. EVs play fundamental roles in cell-cell communication and cell-environment interactions by carrying proteins, nucleic acids such as DNA and RNA (mRNAs, lncRNAs, and miRNAs), and other bioactive molecules that are able to influence the behaviour of recipient cells. EVs have been described as key players in the modulation of tumour progression and the anticancer immune response. In this review, we highlight current knowledge on the role of non-coding RNAs in the modulation of the immune response, focusing on lung cancer. Since EVs are fundamental cell-to-cell mediators, we discuss the current knowledge on the immunomodulatory properties of tumour-derived EVs and, in particular, their ncRNA cargo during the different phases of lung cancer development and progression.

## LUNG CANCER

Lung cancer is the leading cause of tumour-related death worldwide for both men and women^[1]^. Generally, this disease is classified as non-small-cell lung cancer (NSCLC, 85%) and small-cell lung cancer (SCLC, 15%)^[2]^. Histologically, NSCLC is subdivided into squamous cell carcinoma, large cell carcinoma, and adenocarcinoma, which are the most prevalent types^[3]^. For NSCLC, the 5-year survival rate is estimated to be approximately 15% due to late diagnosis, the presence of tumour heterogeneity, and the limited understanding of lung cancer pathogenesis^[4]^. For early-stage NSCLC, surgical resection is the best therapeutic option and is applied alone or in combination with platinum-based chemotherapy, whereas chemotherapy and radiation represent the treatment of choice for advanced or metastatic lung cancer patients. The identification of driver mutations and genetic rearrangements in approximately 50%-60% of NSCLC cases has led to a change in the treatment of subgroups of lung cancer patients with a specific molecular profile^[5,6]^. Recently, improvements were achieved in the management of lung cancer as a result of the development of immune checkpoint inhibitors (ICIs) that block the PD-L1/PD1 axis or CTLA-4^[7]^. Currently, immunotherapy alone or in combination with standard chemotherapy represents a more promising therapeutic option for advanced-stage lung cancer than standard chemotherapy^[8]^. However, no reliable biomarkers are available to stratify patients who will benefit from this therapeutic approach, emphasizing the need to better understand the molecular processes underlying lung cancer development.

The cancer microenvironment has important impacts on the development and progression of lung tumours^[9]^. The tumour microenvironment (TME) includes endothelial cells, cancer-associated fibroblasts, and infiltrating immune cells^[10,11]^. Tumour cells are able to modulate the surrounding environment through the release of several elements, such as cytokines and extracellular vesicles (EVs)^[12]^. EVs can act as mediators of cellular communication through the delivery of their cargoes, such as proteins, lipids, and non-coding RNAs (ncRNAs)^[13,14]^. In this review, we first discuss the role of ncRNAs in the modulation of the immune response in the lung cancer microenvironment and then describe how EVs released from cancer cells modulate the phenotype of infiltrating immune cells to support tumour growth or eliminate tumour cells. Finally, we focus on the importance of ncRNAs carried by EVs from lung cancer cells and their immunoregulatory activity.

### Immune system and lung cancer

Currently, there is a consensus about the importance and clinical relevance of the immune system and cancer interactions during all phases of tumour progression^[15]^. Indeed, the acquisition of oncogenic mutations by non-malignant cells is not sufficient for the full transition to a malignant phenotype. In this regard, several other modifications within the microenvironment are required to fuel cancer cells with nutrients, impair cell death pathways, and, most importantly, help mutant cells escape the control of the immune system^[16]^. Indeed, both the innate and adaptive immune systems can recognize and eliminate cancer cells^[17]^. Normally, the innate immune system, composed of natural killer (NK) cells, polymorphonuclear (PMN) leukocytes, mast cells, and antigen-presenting cells (APCs) such as macrophages and dendritic cells (DCs), is faster than the adaptive immune system in recognizing and eliminating cancer cells through the production of inflammatory cytokines, including interferon-gamma (IFN-γ), and perforin^[18]^. Conversely, adaptive immunity (mainly mediated by T and B cells) takes longer to initiate a response, but it is active after the recognition of specific antigens displayed on the surface of cancer cells, which elicits a more robust and durable anticancer response. However, during cancer progression, cancer cells acquire the capability to avoid immune recognition by adopting different immune escape mechanisms, such as defective processing and MHC class I presentation of cancer-related antigens and the creation of an immunosuppressive microenvironment^[19]^. The latter condition is established through the recruitment of suppressive immune cells, the polarization of immune and stromal cells towards a pro-tumoral phenotype, the production of immunosuppressive cytokines, or the tumour or stromal cell expression of inhibitory immune checkpoint molecules (e.g., CTLA-4 and PD-L1) that can negatively affect the proper functioning of tumour-infiltrating lymphocytes. Together, these alterations strongly impair the immune system, which becomes unable to recognize and eliminate tumour cells, resulting in tumour progression and outgrowth^[20]^.

In the context of the lung cancer microenvironment, two important studies investigated tumour-induced infiltrating lymphoid and myeloid cells and their reprogramming capacity^[21,22]^. Lavin *et al.* described the first innate immune cell atlas of early lung adenocarcinoma lesions, reporting the impaired balance between infiltrating effector CD8^+^ T cells and T regulatory cells (Tregs) at the tumour site, observed as a decline in T cells expressing granzyme B and IFN-γ coupled with an expansion of suppressor T cells^[21]^. While studying the innate immunity compartment, Durrans *et al.* noticed an increased number of bone marrow-derived cells in tumour samples compared to corresponding normal tissue samples^[22]^. In detail, increased production of pro-tumoral factors, mainly osteopontin and the chemokine CCL7, was detected within the tumour microenvironment (TME) and attributed specifically to myeloid cells (both immature monocytic myeloid cells and neutrophils)^[22]^.

Similarly, Lavin *et al.* described several alterations within the TME: the paucity and dysfunction of NK cells, dendritic cells (DCs), and CD16^+^ monocytes, along with an increase in immunosuppressive macrophages^[21]^. In addition, single-cell RNA sequencing revealed that macrophages present within a tumour, which were mainly derived from monocytes with immunosuppressive activity, showed a significantly different transcriptional profile than normal tissue macrophages^[23]^. Interestingly, data from early lung adenocarcinoma showed that tumour-associated macrophages (TAMs) expressed the immunomodulatory transcription factor PPARγ, CD64, CD14, and CD11c and had reduced expression of CD86 and CD206^[21]^.

### ncRNAs and immune regulation in lung cancer

For a long time, proteins were believed to be the only products derived from genetic information having functional significance. For this reason, studying the specific regions of the genome that encode proteins is an appealing field of interest in medical research. Innovative sequencing tools, however, have revealed that the protein-coding region accounts for only 2% of the whole genome and that the remaining 98% encodes thousands of RNA molecules with essential biological and pathological roles as process regulators^[24]^. Historically, these RNAs, known as ncRNAs, were classified into two main categories based on their size: small ncRNAs and long ncRNAs (lncRNAs). Small RNAs are less than 50 nucleotides in length and include microRNAs (miRNAs), ribosomal RNA (rRNA), transfer RNA (tRNA), and piwi-interacting RNA (piRNA). On the other hand, lncRNA segments contain longer sequences, generally exceeding 200 nucleotides, and include pseudogenes and circular RNAs (circRNAs)^[25]^. Among small ncRNAs, miRNAs are the most studied and described in cancer progression^[26]^. MiRNAs are a family of non-coding RNAs composed of 21-25 nucleotides, and their biogenesis is a multistep process that involves the processing of RNA transcripts. MiRNAs are involved in a huge number of functions varying from the transcriptional/post-transcriptional level to the translational level, meaning that miRNAs can regulate a great number of messenger RNAs in a cell^[27]^. It has been proven that a single miRNA can usually modulate several genes and that one gene can be controlled by multiple miRNAs^[28]^. Indeed, this family of ncRNAs is implicated in the regulation of several gene networks through the modulation of oncogenes such as RAS, MYC, and EGFR and tumour suppressors such as TP53, PTEN, and BRCA1^[29]^.

NcRNAs have also been implicated in the regulation of immune cell signalling in lung cancer. The most relevant works describing how ncRNAs modulate immune cell recruitment and functions are summarized in Table 1. A study on NSCLC detected PD-L1 as a downstream target of miR-200/ZEB1, and this targeting contributed to immunosuppression in primary tumour tissue by increasing T-cell exhaustion^[30]^. Moreover, another work by Fujita *et al.* demonstrated the correlation between miR-197 expression and the down-regulation of CKS1B, a key regulator of PD-L1 synthesis^[31]^. On the other hand, miR-3127 was shown to promote PD-L1 overexpression and immune escape in lung adenocarcinoma through STAT3 phosphorylation^[32]^.

**Table 1:** Role of ncRNAs in the regulation of immune response in lung cancer

**References**	**ncRNA**	**Target**	**Function**
Chen *et al.*^[30]^	miR-200	ZEB1 and PD-L1	T cell exhaustion
Fujita *et al.*^[31]^	miR-197	Cyclin-dependent kinase subunit 1 (CKS1B)	Induce tumour progression
Tang *et al.*^[32]^	miR-3127	STAT3/PD-L1	Sustain immune escape
Sun *et al.*^[34]^	lnCRNA XIST	IL-10 and CD163 down-modulation	Conversion to M2-like macrophages
Li *et al.*^[35]^	GNAS-AS1	mir-4319	Promote NSCLC cell growth and metastasis
Tian *et al.*^[36]^	lncRNA HOTAIRM1	HOXA	Increase CD8^+^ cytotoxic T lymphocyte cells
Wu *et al.*^[37]^	circ_0020714	miR-30a-5p/SOX4	Immune evasion and anti-PD-1 resistance
Yang *et al.*^[38]^	CHST1	miR-155 and miR-194	Promote immune escape of lung cancer

ncRNAs: Non-coding RNA s; lncRNAs: long non-coding RNAs; XIST: X-inactive specific transcript; NSCLC: non-small cell lung cancer; PD-1: programmed cell death protein 1, ZEB1: Zinc finger E-box Binding homeobox 1; PD-L1: programmed cell death-ligand 1; STAT3: signal transducer and activator of transcription 3; HOXA1*: *homeobox A1; SOX4: SRY-box transcription factor 4; IL-10: interleukin 10; XIST: X-inactive specific transcript; HOTAIRM1: HOX antisense intergenic RNA myeloid 1; GNAS-AS1: GNAS antisense RNA 1.

Along with miRNAs, lncRNAs have been shown to play a role in the anti-tumour immune response in lung cancer. In a recent article, Sage *et al.* combined single-cell RNA-sequencing data and flow-sorted healthy peripheral blood mononuclear cell (PBMC) data to identify immune-related lncRNAs with the potential to identify infiltrated immune cell populations within tumours^[33]^. Furthermore, considering the role of lncRNAs in the regulation of the expression of oncogenic genes, this information could be correlated with the deregulation of several gene pathways in cancer^[33]^. Sun *et al.* reported that lung cancer cells induced the up-regulation of the lncRNA XIST on macrophages and that this mechanism promoted conversion to an M2-like macrophage phenotype^[34]^. Furthermore, this conversion was characterized by the down-regulation of specific markers such as IL-10 and CD163, which subsequently promoted invasion and migration by lung cancer cells. In this study, the authors proved that the conditioned medium of lung cancer cells induced XIST and promoted the expression of M2-related genes in macrophages^[34]^.

LncRNAs such as GNAS-AS1 were found to regulate the expression of mir-4319 in *in vitro*-differentiated THP-1 macrophages, thus increasing their number and consequently promoting NSCLC cell growth and metastasis^[35]^. In contrast, overexpression of the lncRNA HOTAIRM1 can reduce the immunosuppressive properties of MDSCs. In particular, this lncRNA, through the up-regulation of its target HOXA1, negatively affects the production of immunosuppressive molecules by MDSCs, thus reducing the immune suppression mediated by these pro-tumoral cells^[36]^.

It has been demonstrated that many types of circular RNAs are involved in NSCLC immune evasion. Indeed, circ_0020714 was found to be up-regulated in NSCLC tissues compared with non-tumour adjacent tissues, where it acted as a sponge for mir-30a-5p, which in turn up-regulated the levels of the transcription factor SOX4^[37]^. Furthermore, the circRNA CHST15 has been described to act as an oncogene in lung cancer since its down-modulation correlates with reduced tumour growth. Moreover, CHST15, by sponging miR-155 and miR-194, promotes the expression of PD-L1 on tumoral cells, thus contributing to immune escape during tumour progression^[38]^.

## EXTRACELLULAR VESICLES

“Extracellular vesicles” (EVs) are a generic term describing the lipid-bilayer particles released by almost all cells in the human body. However, these particles are highly heterogeneous, and their classification can differ based on the criteria utilized to differentiate them. EVs originate mainly via two cellular routes, one involving the endocytic cellular pathway and the other involving the plasma membrane. Exosomes, or small EVs, are endosome-derived particles with a mean size of 50-150 nm. Their release relies on the formation of multivesicular bodies in the endosomal compartment and their subsequent fusion with the plasma membrane. In contrast, microvesicles, or ectosomes, have a larger mean size than exosomes (100-1,000 nm), and their formation depends on outwards budding from the plasma membrane^[39]^. Although this classification has been widely used, the cellular origin of these particles remains a challenging issue, as there is no consensus within the EV community on the markers utilized to discriminate different EVs subtypes based on their origin^[40]^. For these reasons, the guidelines of the International Society for Extracellular Vesicles (ISEV) suggest the use of “extracellular vesicles” to indicate isolated particles and the adoption of a classification system based on the physical characteristics of EVs, such as dimension, density, and biochemical properties^[40]^.

Due to their intrinsic properties, EVs act as cellular messengers by carrying different bioactive molecules (proteins, nucleic acids, and metabolites) from one cell to another recipient cell, suggesting their pivotal role in cell-to-cell communication. EVs-related molecules can participate in many biological processes in different pathological conditions^[41]^. In cancer, EVs have been described as mediators in tumour progression-related mechanisms through the modulation of vascular permeability and neoangiogenesis, which allows and supports cell extravasation and metastatic outgrowth. EVs have also been reported to be involved in the modulation of the anticancer immune response^[42,43]^.

### Immunomodulatory functions of tumour-derived EVs

As described in the previous section, an important process in tumour progression is tumour escape: a state where tumour cells, via different mechanisms, prevent their recognition and consequent elimination by immune cells, resulting in tumour growth^[19]^. Among the different signals that can hamper the proper functioning of immune cells are interactions with tumour-derived EVs (tEVs)^[44,45]^. In particular, tEVs can have opposite effects: while they can suppress immune cell function, they can also express different tumour antigens with immunogenic properties on their surface^[46] ^[Table 2].

**Table 2:** Immunomodulatory properties of tumour derived-EVs

**References**	**EVs origin**	**Specimens**	**Isolation method**	**tEVs effective molecule**	**Functional role**
	**Pro-tumoral immune response**				
Chen *et al.*^[48]^	Melanoma	cell lines	Ultracentrifugation	PD-L1	Inhibition of T cells proliferation and functionality
Ohue *et al.*^[50]^	Melanoma	cell lines	Capture beads	Induction of TLR3-TRIF signaling in DCs with IFN-β production	Increased number of tumour-infiltrating Treg and tumour outgrowth
Nakazawa *et al.*^[51]^	Leukemia	cell lines and plasma	Ultracentrifugation for cell lines and SEC for plasma samples	4-1BBL/CD137L molecules	Activation of Treg cells
Haderk *et al.*^[53]^	B-chronic lymphocytic leukaemia	cell line	Ultracentrifugation and sucrose density cushion	Non-coding Y RNA hY4	Induction of PD-L1 expression on monocytes
Vignard *et al.*^[54]^	Melanoma	cell lines	Ultracentrifugation and ExoQuick®	miR-3187-3p, miR-498, miR-122, miR-149, and miR-181a/b	Reduced TCR signaling pathway and cytotoxic activity in CD8^+^ T cells
Shinohara *et al.*^[55]^	Colorectal cancer	cell lines	Ultracentrifugation	miR-145	M2-like polarization via histone deacetylase 11 down modulation
Xun *et al.*^[56]^	Breast carcinoma	cell lines	Ultracentrifugation	miRNA-138-5p	M2-like polarization via H3K27 histone demethylase KDM6B inhibition
Zhang *et al.*^[57]^	Glioblastoma and microglia	cell lines	Ultracentrifugation	circular RNA circ_0012381, miR-340-5p	Increased secretion of CCL2 by microglia cells that in turn promotes tumour growth
	**Antitumoral immune response**				
Menay *et al.*^[58]^	T lymphoma	mouse model	Sucrose density cushion	CD24 and Hsp90	Generation of specific humoral and cellular immune response
Daßler-Plenker *et al.*^[59]^	Melanoma	cell lines	Ultracentrifugation	NKp-30 ligands (BAG6, BAT3)	Activation of the cytotoxic activity of NK cells via NKp-30 receptor
Ma *et al.*^[60]^	Melanoma	cell lines	Ultracentrifugation	TAAs	Promotion of MHC class I: TAAs complex formation in DC

EVs: Extracellular vesicles; tEVs: tumour-derived EVs; PD-L1: programmed cell death-ligand 1; TLR3: toll-like receptor 3; DCs: dendritic cells; miRNAs: microRNAs; TAAs: tumour associated antigens; MHC: major histocompatibility complex; NK: natural killer; CCL2: chemokine C-C motif ligand 2; KDM6B: histone lysine demethylase 6B; TCR: T cell receptor.

A well-described process through which tEVs inhibit the functions of immune cells is the expression of immunoregulatory molecules, such as PD-L1, on their surface^[47]^. PD-L1 on melanoma-derived EVs was observed to inhibit the proliferation and cytotoxic activity of CD8^+ ^T cells *in vitro*^[48]^. The suppression mediated by PD-L1-EVs required an interaction between ICAM-1, which is up-regulated together with PD-L1 following IFN-γ stimulation, and LFA-1, which is expressed on activated T cells. Indeed, the blockade of ICAM-1 on EVs prevented the interaction of melanoma-EVs with CD8^+^ T cells and the consequent inhibition mediated by PD-L1^[49]^. Notably, the immunosuppressive properties of EVs expressing PD-L1 were demonstrated in models involving EVs from tumour cell lines, which could differ from patient-derived EVs and their real anti-immune activity.

CD4^+^ Tregs represent important helper cells involved in tumour growth since they down-regulate the cytotoxic antitumour activity of CD8^+^ T cells, and their presence within a tumour correlates with a poor prognosis in different cancer types^[50]^. In melanoma-bearing mice, the internalization of tEVs by DCs was shown to stimulate IFN-β production via the endosomal TLR3 signalling pathway, resulting in an increased number of Tregs and tumour outgrowth^[51]^. Leukaemia-derived EVs (obtained from either human cell lines or patient plasma) that transport 4-1BBL/CD137L molecules were shown to induce activation and effector phenotypes in Tregs via the upregulation of CD39 and TNFR2 expression^[52]^.

Impairment of immune function can be achieved by other mechanisms that involve several types of non-coding RNA. In chronic lymphocytic leukaemia, the non-coding Y RNA hY4 enriched in tumour exosomes induces the expression of PD-L1 on monocytes via stimulation of endosomal TLR7 signalling, thus promoting tumour escape^[53]^. In contrast, the TCR and TNF-α signalling pathways in CD8^+^ T cells are disrupted by the activity of miR-3187-3p, miR-498, miR-122, miR-149, and miR-181a/b delivered by melanoma-derived EVs^[54]^. In addition, non-coding RNAs associated with tEVs also influence the polarization of tumour-infiltrated macrophages towards an M2 phenotype, which is fundamental for tumour progression. For instance, in colorectal cancer, this polarization occurs via the down-modulation of histone deacetylase 11 mediated by miR-145 within colorectal cancer cell-derived EVs^[55]^. In addition, miR-138-5p, which has been observed in breast cancer-derived EVs, induces an M2-like phenotype in macrophages and promotes tumour growth by inhibiting the H3K27 histone demethylase KDM6B^[56]^. On the other hand, in brain malignancies, the uptake by microglia of EV-sorted Circular RNAs (circRNA) circ_0012381, which sponges with miR-340-5p, increases ARG1 expression, resulting in CCL2 secretion, which in turn promotes the growth of glioblastoma cells^[57]^.

On the one hand, tEVs are able to affect immune function negatively, as largely discussed above; on the other hand, the same tEVs can elicit a response against tumours by stimulating different immune populations. Indeed, tEVs obtained from the ascites of T-cell lymphoma-bearing mice expressed CD24 and Hsp90, malignant markers, on their surface and induced a competent immune response resulting in the rejection of a subsequent tumour challenge in syngeneic naïve mice^[58]^. Daßler-Plenker *et al.* showed that stimulation of the cytosolic immune sensor RIG-I in melanoma cells affected the protein surface expression of tEVs, promoting NK cell functionality^[59]^. tEVs can also positively affect the presentation of tumour associated antigens (TAAs) by DCs and thus have an intrinsic potential as a vaccine. For instance, the endocytosis of melanoma-derived microparticles efficiently promoted the formation of MHC class I-tumour antigen complex together with the induction of the costimulatory molecules CD80 and CD86. The concomitant expression of these molecules with MHC complexes allowed a highly efficient tumour antigens presentation to CD8^+^ T cells^[60]^.

EVs released by infiltrating Treg cells could prevent the proper functioning of other T-cell subtypes. Additionally, let-7d in exosomes is transferred to Th1 cells, contributing to immune cell suppression, which demonstrates that within the tumour microenvironment, tumour cells secrete EVs to influence the behaviour of immune cells^[61]^.

### Immune cell regulation by non-coding RNA in lung cancer-derived EVs

**Figure 1:**
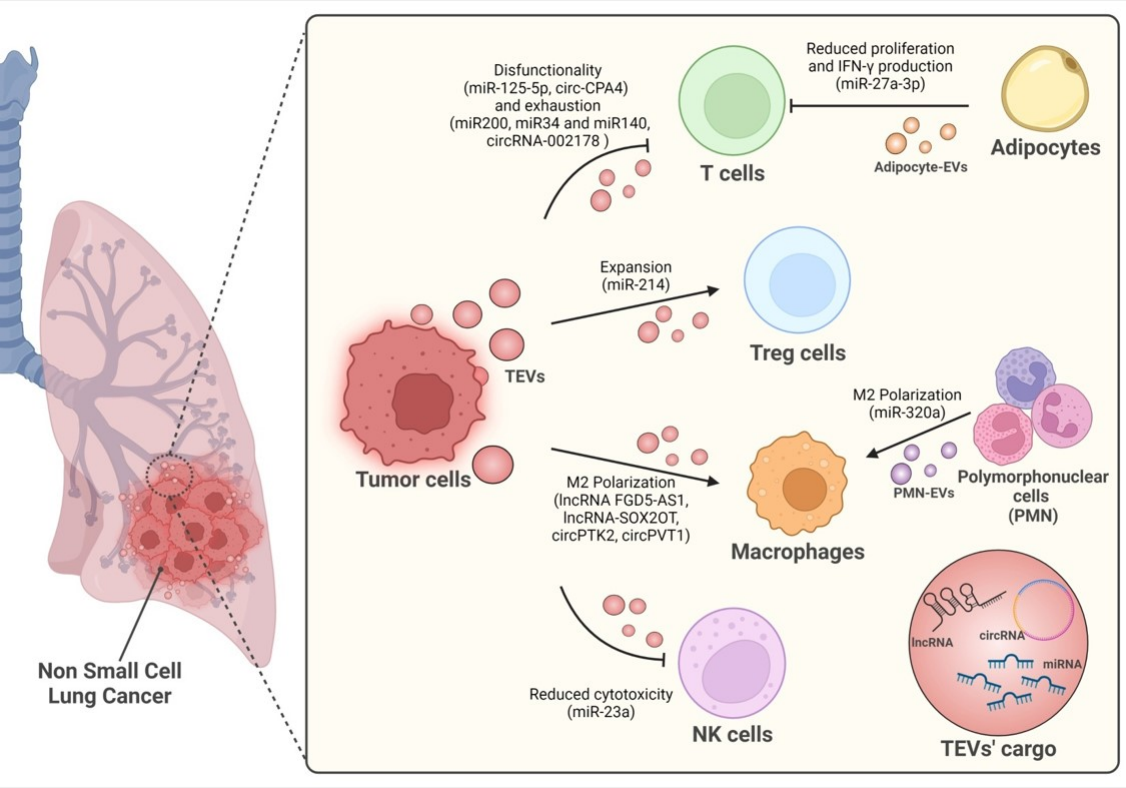
Immune cells regulation of non-coding RNA inside lung cancer derived-EVs.

The EV field has widely expanded in recent years, but only a few studies have aimed to understand the role of EVs in immune regulation in the context of lung cancer. Hereafter, we report all the articles showing a link between EV-contained non-coding RNAs and immune regulation in lung cancer. Figure 1 highlights the key roles played by tEVs in affecting the behaviours of different cell populations within the TME and the many different ncRNAs involved in these processes.

#### MicroRNAs

Hypoxia is one of the most important drivers of lung cancer progression. Indeed, the hypoxic tumour microenvironment strongly affects the release of tEVs^[62]^. Furthermore, hypoxic tEVs enriched in TGF-β and miR-23a impair NK cell cytotoxic abilities by down-regulating two fundamental receptors of NK cell activation and degranulation (NKG2D and CD107a)^[63]^.

Interestingly, the level of adipocyte-derived miR-27a-3p was observed to decrease as body mass index (BMI) increased, and this was inversely correlated with the level of the costimulatory gene *ICOS, *which is important in T-cell activation^[64]^. Although the link between miR-27a-3p and the *ICOS* gene was not directly demonstrated, *in vitro* experiments showed that EVs from adipocytes silenced for miR-27a-3p displayed higher levels of ICOS^+^ T cells and higher levels of IFN‐γ production. Similarly, Peng *et al.* observed a correlation between the up-regulation of EV-miR-125b-5p and T-cell dysfunction at baseline in nonresponsive NSCLC patients undergoing ICI therapy^[65]^. Moreover, they identified three miRNAs from the miR-320 family (miR-320d, miR-320c, and miR-320b) associated with a poor prognosis and response to ICIs, identifying these miRNAs as potential biomarkers for therapy response^[65]^. In addition, our group described circulating miR-320a shuttled by PMN-derived EVs in high-risk heavy smokers, defining its critical role in the induction of a pro-tumorigenic M2-like phenotype in macrophages via STAT4 targeting^[66]^.

An interesting study on T-cell modulation highlighted the role of the miR-200/ZEB1 axis in the modulation of the levels of PD-L1 on lung tumour cells and, consequently, in T-cell exhaustion^[30]^. A similar role was also attributed to miR-34 and miR-140, which both directly bind PD-L1 in NSCLC cells^[67,68]^. Furthermore, Treg cell expansion could also be mediated via the regulation of PTEN by miR-214 carried within microvesicles released by different types of cancer, highlighting a possible common mechanism to induce tumour progression^[69]^.

#### Long non-coding RNAs

Similar to the members of the ncRNA family, lncRNAs have been observed to be deregulated in all stages of lung cancer development^[70]^. In 2016, Wang *et al.* first reported the involvement of EV-related lncRNAs in lung cancer by highlighting a new mechanism of interaction between lung tumour cells and their microenvironment^[71]^. Indeed, EVs produced by lung tumour cells were found to be responsible for a deep alteration in the lncRNA profile in mesenchymal cells. Even if a direct association with lncRNAs in tEVs was not provided, this study described for the first time the role of microenvironmental lncRNA perturbation in lung cancer^[71]^.

Within the NSCLC microenvironment, TAMs are one of the main cellular components: they directly support cancer cell growth, survival, invasion, and metastasis and additionally provide protection to NSCLC cells via immune evasion strategies^[72]^. Recently, evidence suggesting EV-mediated crosstalk between lung tumour cells and macrophages was reported^[73]^. Indeed, in lung cancer, the lncRNA FGD5-AS1 detected in tEVs was found to be responsible for phenotypic alterations in macrophages, which resulted in the upregulation of genes involved in M2 polarization^[74]^. Interestingly, tEV-lncRNA-SOX2OT was detected in the blood of NSCLC patients and linked to the formation of pro-metastatic features by targeting the miRNA-194-5p/RAC1 signalling axis in osteoclasts^[75]^. Indeed, SOX2OT was detected inside EVs from NSCLC cells and associated with the induction of an M2-like phenotype and concomitant M1 polarization inhibition through the miR-627-3p/SMAD signalling pathway, resulting in increased EGFR-TKI resistance.

#### Circular RNAs

Circular RNAs (CircRNAs) are an emerging field in cancer research, especially in NSCLC, as they were demonstrated to play pivotal roles in carcinogenesis, tumour formation, proliferation, migration, invasion, and sensitivity to therapy^[76]^. The first evidence of the presence of circRNA in cancer EVs was reported in 2015 when Li *et al.*, using RNA-seq methods, demonstrated the enrichment of circRNAs in tEVs compared to the cell of origin^[77]^. However, although many efforts have been made in recent years to understand the role of circRNAs in cancer progression, their impact on NSCLC has not been investigated as carefully as that of other types of non-coding RNAs. Most studies on EV-associated circRNAs in lung cancer aimed to comprehend their role in tumour cells better; thus, their involvement in the modulation of the immune landscape is still unknown^[78]^.

Interestingly, a multifaceted role for the circ-CPA4/let-7 miRNA/PD-L1 axis in NSCLC was described by Hong *et al.*, showing how circ-CPA4 promoted the production of tumoral-PD-L1^+^-EVs, which interacted with T cells to establish CD8^+^ T-cell inactivation, tumour immune escape and resistance to chemotherapy^[79]^. Similar results were obtained by Wang *et al.*, who demonstrated the presence of high levels of circRNA-002178 in tumour samples and lung cancer cell lines and showed that enhancing PD-L1 expression led to T-cell exhaustion^[80]^. Importantly, the authors showed that circRNA-002178 was also present in the plasma-EVs of NSCLC patients and that its delivery into CD8^+^ T cells induced PD1 expression. Regarding the interplay between circRNA-EVs and innate immunity in lung cancer, only a few studies have described the involvement of circRNA-EVs in modulating macrophage polarization. Interestingly, circPTK2 was observed to be highly expressed in lung cancer patient serum EVs and correlated with the cancer stage. Most importantly, macrophages enriched in circPTK2 were found to be relatively pro-tumoral (M2 polarization), suggesting a possible role for circPTK2 in the EV-mediated crosstalk between cancer cells and the stroma^[81]^. Another circRNA linked to macrophage polarization is circPVT1, which was observed in EVs from lung cancer patients (blood) and cell lines. Indeed, the delivery of circPVT1 to macrophages via EVs was shown to cause M2-like polarization by sponging miR-124 and consequently increasing EZH2 expression. Moreover, the authors showed that co-incubation with EV-treated macrophages prompted lung cancer cell proliferation, migration, and invasion^[82]^. Taken together, these studies suggest a potential role for EV-circRNA in the modulation of the immune microenvironment. There is still much work to be done to better elucidate the involvement of circRNA-EVs and immune modulation in lung cancer.

## CONCLUSION

Extracellular vesicles as modulators of the immune response are still an expanding area of research. Here, we described several studies showing significant roles for these particles as diagnostic or prognostic biomarkers in cancer. However, to reach clinical implementation, several challenges still need to be addressed.

A consensus still needs to be reached among researchers regarding the term “extracellular vesicles” and their utilization, although the ISEV stated its agreement for the use of the term when indicating lipid-bilayer particles released by cells. The inappropriate use of “exosomes” and “microvesicles” creates confusion and misunderstanding among readers^[40]^. Another issue to address is EV characterization: the majority of the studies investigating the role of EVs in cancers are poorly characterized, as illustrated by the minimal information for studies of extracellular vesicles (MISEV) guidelines^[40]^. Lack of adherence to these guidelines affects the quality of published findings and the reproducibility of results.

Notably, the immunoregulatory function of tumour-derived EVs has mostly been demonstrated using EVs separated from the conditioned media of tumour cell lines. This approach is completely different from using circulating patient-derived EVs obtained from blood, which are mainly derived from other types of cells. Indeed, all results should be confirmed using cancer patient samples, in which tEVs are present along with EVs of different cellular origins. This would allow us to comprehend whether the role of EVs is strictly correlated to the tumour microenvironment or at the systemic level and, therefore, relevant to immune regulation.

In the last twenty years, non-coding RNAs have emerged as reliable candidates for predictive and prognostic biomarkers and therapeutic targets in cancer. However, implementation of these small molecules in the clinical setting has yet to be ready due to several methodological issues that need to be addressed. In this regard, standardized procedures for ncRNA isolation and detection should be established among researchers to avoid inconsistencies and lack of reproducibility among different studies. Nonetheless, a better comprehension of the origin and mechanisms of release of these molecules is necessary before they are implemented in the clinical setting. Despite these challenges, EVs and their non-coding RNA cargo could represent an interesting tool for cancer treatment management.

To date, research has unveiled pivotal functional EV-related ncRNAs involved in modulating the tumour-immune relationship and suggested their potential value involvement in monitoring and predicting treatment responses in lung cancer patients.

## DECLARATIONS

### Copyright

© The Author(s) 2023.
